# Whole-Genome Sequencing of Three *Lactiplantibacillus plantarum* Strains Reveals Potential Metabolites for Boosting Host Immunity Safely

**DOI:** 10.4014/jmb.2402.02013

**Published:** 2024-07-31

**Authors:** I-Chen Li, Yueh-Lun Lee, Tsung-Ju Li, You-Shan Tsai, Yen-Lien Chen, Chin-Chu Chen

**Affiliations:** 1 Biotech Research Institute, Grape King Bio Ltd, Taoyuan City 325, Taiwan; 2 Department of Microbiology and Immunology, School of Medicine, College of Medicine, Taipei Medical University, Taipei City 110, Taiwan; 3 Department of Food Science, Nutrition, and Nutraceutical Biotechnology, Shih Chien University, Taipei City 104, Taiwan; 4 Institute of Food Science and Technology, National Taiwan University, Taipei City 106, Taiwan; 5 Department of Bioscience Technology, Chung Yuan Christian University, Taoyuan City 320, Taiwan

**Keywords:** *Lactiplantibacillus plantarum*, safety evaluation, immunomodulation, cytokines, secondary metabolites, bioinformatics

## Abstract

In response to the growing demand for immune-related products, this study evaluated the safety and immune-modulating potential of three newly discovered *Lactiplantibacillus plantarum* strains (GKM3, GKK1, and GKD7) through toxicity tests and whole-genome sequencing. Safety evaluations, including the analysis of antimicrobial resistance genes, virulence factors, plasmids, and prophages, classified these strains as safe for human consumption. Acute oral toxicity tests further supported their safety. To evaluate their immune-modulating potential, dendritic cells were exposed to these strains, and the secretion of key cytokines (IFN-β and IL-12) was measured. Among the strains, GKK1 exhibited the highest enhancement of IFN-β and IL-12 production, suggesting its potential as an immune-stimulating probiotic. Bioinformatics analysis revealed potential metabolic pathways and secondary metabolites, including predicted bacteriocins, associated with immune modulation. The presence of a nitrate reductase region in the GKK1 strain indicated its ability to produce nitric oxide, a critical molecule involved in immune regulation and host defense. The presence of glucorhamnan-related gene clusters in GKK1 also suggested immune-enhancing effects. Nitrate reductase expression was confirmed using qPCR, with the highest levels detected in GKK1. Moreover, this study is the first to show an anti-inflammatory effect of plantaricin A, linked to its presence in strain GKM3 and its potential therapeutic applications due to sequence similarity to known anti-inflammatory peptides. Overall, these three *L. plantarum* strains demonstrated a safe profile and GKK1 showed potential as an immunity-enhancing probiotic. However, additional investigation is required to confirm the involvement of specific metabolic pathways, secondary metabolites, and bacteriocins in immune responses.

## Introduction

From the moment of birth, the human body becomes home to a vast array of microorganisms, which inhabit various areas like the skin and mucosal surfaces, including the gastrointestinal tract [1]. These microorganisms, which encompass fungi, viruses, and parasites, form what is known as the microbiome and function as a cohesive unit within the human host, contributing to numerous vital processes such as circadian rhythmicity, nutritional responses, metabolism, and immunity [2]. In a healthy state, the gut microbiota is balanced [3]. However, during times of illness, the gut microbiota becomes imbalanced, resulting in dysbiosis [4]. Previous studies have shown that changes to the gut microbiota or dysbiosis can result in pathogen invasion and increase the risk of obesity, type 2 diabetes, inflammatory bowel disease, cancer, and cardiovascular, liver, and central nervous system disorders [5]. Therefore, maintaining a favorable balance of gut microbiota is important for the health of the host.

Probiotics are a group of live microorganisms that provide health benefits to the host when administered in appropriate doses [6]. They are widely known to maintain and restore gut homeostasis, which plays a significant role in the body's resilience by regulating the development and function of immune cells [7]. Studies have shown that the components or metabolites of probiotics can act as microbe-associated molecular patterns (MAMPs) and bind to pattern recognition receptors (PRRs) found on innate immune cells, such as macrophages and natural killer (NK) cells, to have either pro- or anti-inflammatory effects on the host [8]. Interestingly, recent studies have introduced the concept of innate immune memory, which refers to the epigenetic changes that macrophages or NK cells undergo in response to an initial stimulus and how it can make these cells more responsive (trained innate immunity) or less responsive (tolerance) to a subsequent stimulus [9]. Considering that these innate cells can produce stronger or diminished responses to future pathogen exposure, using probiotics as epigenetic adjuvants could be particularly advantageous in providing enhanced antiviral resistance for a limited period, such as during a pandemic like COVID-19, in a susceptible population.

However, research has shown that the effectiveness and safety of probiotics can vary depending on strains and cannot be generalized [10]. Therefore, it is important to thoroughly examine the risk factors associated with each individual strain. The main objective of this study was to systematically assess the safety of three newly identified *Lactiplantibacillus plantarum* strains (GKM3, GKK1, and GKD7) from Taiwan through genomic and oral toxicity analysis. Subsequently, the ability of these selected *L. plantarum* strains to stimulate pro-inflammatory cytokines in co-culture with dendritic cells was evaluated. Then, we utilized a variety of computational genome mining tools. We made comparisons between the strains to predict the organization of bacteriocin cluster genes, as well as other primary and secondary metabolites that could potentially enhance the immune response. Finally, we confirmed strain-specific genes identified through comparative genomic analysis using RT-PCR. Additionally, we validated the anti-inflammatory activities of strain-specific metabolites using LPS-induced RAW 264.7 macrophage cell platforms. These findings will confirm the effectiveness and safety of these three selected novel *L. plantarum* strains as immunomodulatory probiotics.

## Materials and Methods

### Preparation of Samples

*Lactiplantibacillus plantarum* GKM3, GKK1, and GKD7 strains were isolated from pickled mustard green, pickled chili, and Taiwanese kimchi, respectively, by Grape King Bio Ltd., located in Taoyuan City, Taiwan. Based on the results of an internal experiment monitoring interleukin-6 variations in RAW 264.7 macrophage cells, strains GKK1, GKD7, and GKM3 were chosen due to their distinct higher (GKK1 > 50 pg/ml) and lower (GKD7 and GKM3 <10 pg/ml) immune-stimulating effects. These strains were authenticated and preserved at the Bioresource Collection and Research Center (BCRC) of the Food Industry Research and Development Institute (FIRDI) with the preservation numbers of BCRC 910787, 910919, and 910877, respectively. To activate the selected strains from their glycerol storage, they were first cultured in 20 ml of MRS media at 37°C (Difco, USA) and then subcultured into a 2 L flask containing 1.2 L of MRS broth. The cultures were incubated at 37°C for 16 h. Subsequently, the cultures were scaled up to 80% of the working volume, which is equivalent to 12 tons, in a 15-ton fermentor using synthetic medium (5% glucose, 2% yeast extract, 0.05% MgSO_4_, 0.1% K_2_HPO_4_, and 0.1% Tween 80, pH 6.0) at 37°C for 18 h to show the potential for mass production and commercialization of these strains. For in vitro cell culture, the final liquid cultures were adjusted to pH 7 and two concentrations of probiotics (dendritic cells: bacteria ratio; 1:10 and 1:100, each adjusted to add 10 ul/well) were added to primary murine bone-marrow derived dendritic cells and RAW 264.7 (TIB-71; ATCC, USA) cells. For animal studies, the final liquid culture was freeze-dried using a freeze dryer (FD50L-8S-S; KingMech, Taiwan) into a powder with a mean concentration of at least 5 × 10^11^ CFU/g.

### Complete Genome Sequencing and Comparison Genomics

Total genomic DNA from 20 ml samples was extracted using the QIAamp PowerFecal Pro DNA Kits following the manufacturer's instructions (Qiagen, Germany). A size selection was performed using the KAPA Hyper Beads (KAPA Bio-systems, USA) in a 0.4x ratio to enrich DNA fragments larger than 3 kb. The DNA concentration was determined using a Qubit 4.0 fluorometer (Thermo Fisher Scientific, USA), and the fragment size was monitored using the Qsep 100TM system (BiOptic, Taiwan). The long-read sequencing libraries were constructed through several steps including end repair, A-tailing, barcoding, and adapter ligation. In summary, 1 μg of DNA intended for library preparation underwent end repair and A-tailing using the 1D ligation-based sequencing kit (SQK-LSK-109; Oxford Nanopore, UK). A unique dT-tailed barcode adapter was then ligated onto the dA-tailed template with the Native Barcoding Expansion kit (EXP NBD104; Oxford Nanopore). The libraries were then sequenced using a PromethION Flow Cells (FLO-PRO002; Oxford Nanopore) on a PromethION 24 device following the manufacturer's recommendations.

Raw sequencing data were decoded using Guppy (v5.0.7) with the High Quality basecalling mode [11]. Reads with an average quality above Q9 were considered as "pass" reads for subsequent analysis. The sequencing results were then checked by Nano Pack (v1.1.0) to validate the read length profile [12]. Raw reads were assembled using Flye (v2.8.3) [13], polished with Racon (v1.4.22) [14], and aligned by Minimap2 (v2.17) [15] with default parameters. Finally, the remaining systematic errors were polished using Medaka (v1.2.3) [16] and Homopolish (v0.2) [17]. The fully polished contigs were analyzed using QUAST (v5.0.2) [18] and BUSCO (v5.0.0) [19] to evaluate the quality of the assemblies and the completeness of the genome, respectively.

To evaluate the genetic relatedness, the average nucleotide identity (ANI) was calculated using the JSpecies webserver (https://www.ribocon.com/jspeciesws.html, accessed on 20 Nov 2023) [20] among GKM3, GKK1, GKD7, and a reference strain (*L. plantarum* ATCC 14917/DSM20174 [21]). The PanExplorer web server (http://panexplorer.southgreen.fr, accessed on 20 Nov 2023) [22], an online tool hosting a suite of advanced tools including PGAP [23], Roary [24], PanACoTA [25] and RPSblast against the COG database [26], was employed for the analysis and comparison of complete bacterial genomes. The whole-genome sequences of three *L. plantarum* strains have been deposited at the GenBank repository under the BioProject accession number PRJNA1051836 (accession numbers SAMN38800459, SAMN38800458, and SAMN38800457 for strains GKK1, GKD7, and GKM3, respectively).

### Genomic Aspects Related to Food Safety- Antibiotic Resistance Genes, Virulence Factors, Pathogenic Genes, Plasmids, and Prophages

The probiotic potential risk score (PPRS) [27] (classified as low-risk (≤ 4), medium-risk (4-6), and high-risk (≥ 6)) for *L. plantarum* GKM3, GKK1, and GKD7 was determined by analyzing the complete nucleotide sequence using ProbioMinServer [28] (https://probiomindb.imst.nsysu.edu.tw/index.php, accessed on 20 Nov 2023), which utilizes selected tools recommended by the European Food Safety Authority (EFSA) guidance [29]. The analysis included assessing antibiotic resistance genes (ARGs) (Resistance Gene Identifier v6.0.0 software searches against the Comprehensive Antibiotic Resistance Database (CARD) Variants v4.0.0 [30], ResFinder v4.0 [31], and AMRFinderPlus v3.10.42 [32]), virulence factors (VFs) (BLASTN v2.8.1+ searches against the setB database of the pathogenic Virulence Factor Database (VFDB) (June 2022 release) [33] and VirulenceFinder v2.0.3 [34]), pathogenic genes (PGs), plasmids, and prophages.

### Oral Acute Toxicity Study

A total of 20 male and 20 female eight-week-old Sprague-Dawley rats weighing 270 ± 20 g were obtained from BioLASCO Taiwan Co. (Taiwan). They were kept in polypropylene cages (*n* = 2) and housed in a temperature-controlled room at 23 ± 2°C with a 12-h light-dark cycle. The rats were provided with unlimited access to standard rodent diet (1324N; Altromin, Germany) and purified water. The acute toxicity test was conducted following the method described in OECD Guideline 423 [35]. After a 7-day acclimatization period, rats of the same sex were randomly divided into four groups, with five rats in each group. The control group received a single dose of 10 ml normal saline, while the three treatment groups each received a single dose of 2000 mg/kg/day *L. plantarum* in 10 ml of normal saline. This dose is equivalent to the human dose, calculated based on body surface area using the formula provided by the US Food and Drug Administration, which corresponds to 1.56 × 10^12^ CFU/day for a human weighing 60 kg [36]. The rats were individually monitored for signs of toxicity, mortality, morbidity, and changes in body weight at 30 min, 4 h, and continuously up to day 7 after dosing. On day 8, all overnight fasted rats were euthanized using carbon dioxide anesthesia, and blood and organs were collected for hematological, clinical, biochemical, and histopathological examination. This study was approved by the Institutional Animal Care and Use Committee at SuperLab in Taiwan (No. 108-1l).

### Immunomodulation of Dendritic Cells by *L. plantarum* Strains

Animal care and handling protocols were approved by the animal use committee of the College of Medicine, Taipei Medical University (No. LAC-2021-0228). Bone marrow cells were collected from 5-week-old C57BL/6 mice (National Laboratory Animal Center, Taiwan) and cultured in RPMI-1640 (Thermo Fisher Scientific) with 5% fetal bovine serum (Thermo Fisher Scientific) and 500 U/ml granulocyte-macrophage colony-stimulating factor (PeproTech, USA) for 6 days, as previously described [37]. On day 7, bone marrow-derived dendritic cells (105 cells/well) were cultured in 48-well plates and treated with different concentrations (dendritic cells: bacteria ratio; 1:10 and 1:100) of *L. plantarum* GKM3, GKK1, GKD7 or positive control (100 ng/ml LPS (Sigma-Aldrich, USA) and 250 multiplicities of infection (MOI) adenovirus (generated by homologous recombination and amplified in 293 cells as previously described [38]) for IL-12 and IFN-β, respectively) for 24 h. After incubation, culture supernatants were collected and pooled triplicates. The quantities of cytokine IL-12 (#BMS6004; Thermo Fisher Scientific) and IFN-β (#42400-1; R&D Systems, USA) were then determined using commercially available enzyme-linked immunosorbent assay (ELISA) kits, following the manufacturer's instructions.

### Prediction of Bacteriocins and Primary and Secondary Metabolites

The BAGEL4 web server was used to detect bacteriocins (http://bagel4.molgenrug.nl/, accessed on 20 Nov 2023) [39]. To detect potential primary and secondary metabolite biosynthesis gene clusters, both gutSMASH v1.0.0 (https://gutsmash.bioinformatics.nl/, accessed on 20 Nov 2023) [40] and antiSMASH v6.0.0 (http://antismash.secondarymetabolites.org, accessed on 20 Nov 2023) [41] web servers were employed with default and strict parameters, respectively.

### Total RNA Isolation and Quantitative PCR (qPCR) Analysis

Total RNA from samples was extracted using the Quick-RNA Fungal/Bacterial Kit in accordance with the manufacturer's instructions (Zymo Research, USA). Subsequently, 500 ng of the isolated RNA was reverse-transcribed into cDNA using the iScript cDNA Synthesis Kit (Bio-Rad, USA) to generate cDNA. Reactions were conducted on the CFX Connect Real-Time PCR Detection System (Bio-Rad) utilizing previously reported primer sets (Table 1) [42, 43]. The nitrate reductase primer, chosen based on a prior study [44] that utilized an extensive range of primer sets to target the nitrate reductase gene, showed a significant correlation between gene abundance and potential denitrification activity. Thereafter, the relative mRNA expression level was normalized to 16S rRNA expression, and quantification was achieved via the ΔΔCt method.

### Anti-Inflammatory Activities

RAW 264.7 macrophages (ATCC) were cultivated at a density of 2.5 × 10^5^ cells/ml in a 96-well plate. These cells were pretreated with varying doses of plantaricin A (sourced from NovoPro Bioscience Inc., China) at concentrations of 1, 5, and 25 μM, for an hour prior to being stimulated with 100 ng/ml of LPS for a duration of 24 h. Following incubation, the cells underwent centrifugation and the supernatant was collected. Cell viability was then assessed via the MTT assay [45]. Subsequently, the concentrations of interleukin-6 (#BMS603-2; Thermo Fisher Scientific) and tumor necrosis factor-alpha (#BMS607-3; Thermo Fisher Scientific) in the cell culture supernatants were measured using an ELISA kit, in accordance with the manufacturer's instructions.

### Statistical Analysis

All values were presented as mean ± the standard deviation (SD). For in vivo studies, statistical differences were determined using one-way ANOVA followed by Duncan’s multiple range test using SPSS Statistics v22 (IBM Corp., USA). For in vitro studies, statistical significance was determined using one-way ANOVA with Tukey's post-test.

## Results

### General properties and Comparative Genomic Analysis of *L. plantarum* GKM3, GKK1, and GKD7 Strains

**Fig. 1:**
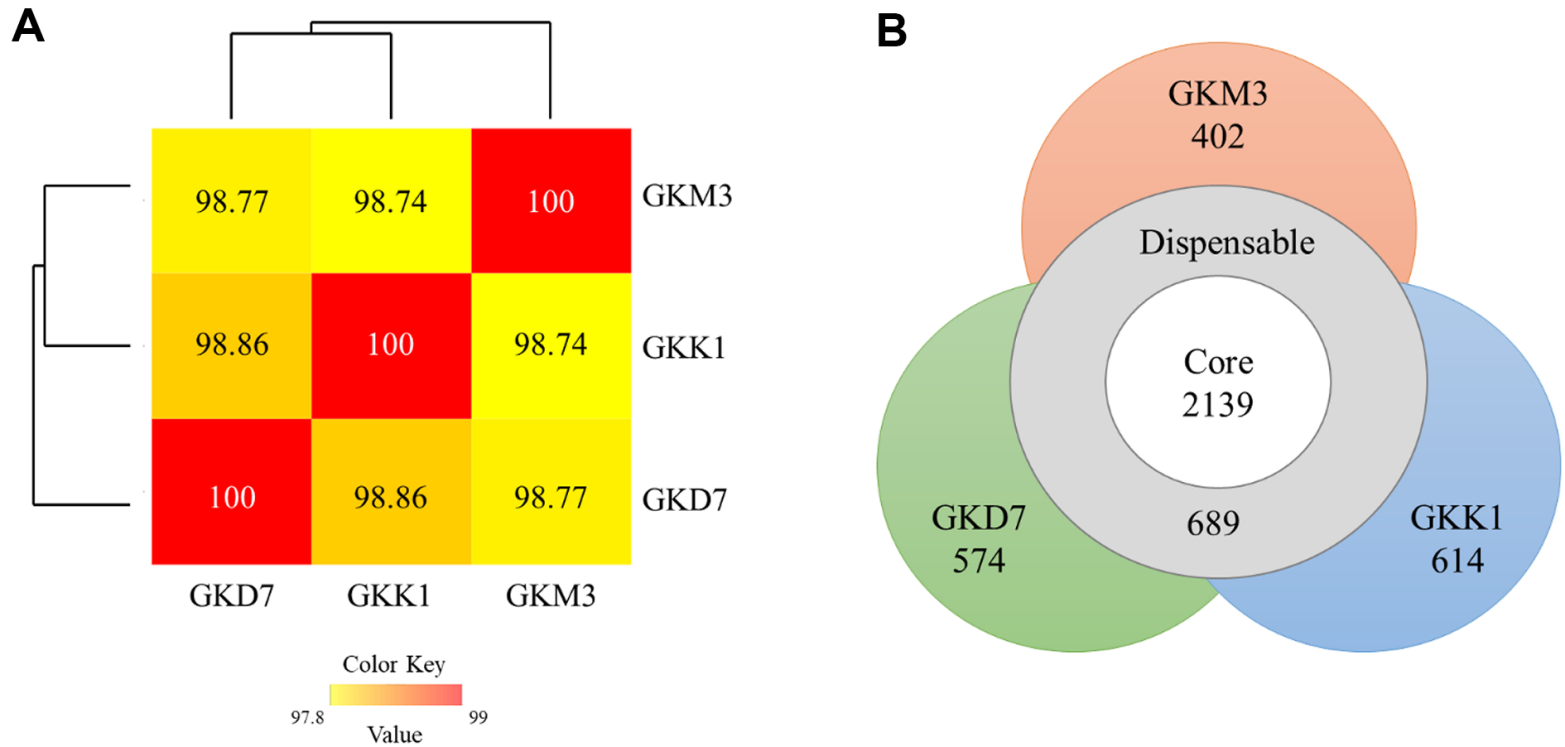
Genomic comparison of *L. plantarum* strains GKM3, GKK1, and GKD7 based on (A) average nucleotide identity (ANI) and pan-genome analysis.

The general genome features of *L. plantarum* strains GKM3, GKK1, and GKD7 are presented in Table 2. These three strains have an average length ranging from 2.99-3.10 Mbp, 2958-3258 coding sequences (CDS), and a GC content of approximately 45%. While the number of rRNA genes is similar, the number of tRNA genes varies slightly among these three strains (68, 63, 67 for strains GKD7, GKK1, and GKM3, respectively). The genetic relatedness among these strains was evaluated by calculating the average nucleotide identity (ANI) (Fig. 1A) and performing a pan-genome analysis, providing insights into their evolutionary relationships and functional capabilities. The ANI values based on BLAST for GKM3, GKK1, and GKD7 were 98.58%, 98.82%, and 98.91%, respectively, when compared to a type strain of a similar species (*L. plantarum* ATCC14917) (data not shown). These values suggest that they share a common ancestor (≥ 95–96% used as a criterion). In terms of pan-genome analysis, the results indicate that these three strains have 2139 core genes (present in all strains), 689 dispensable genes (found in some but not all strains), and 1590 strain-specific genes (unique to a single strain) (Fig. 1B). Based on these findings, it can be expected that these three *L. plantarum* strains share basic probiotic characteristics while also exhibiting some distinct characteristics.

### Safety Assessment of *L. plantarum* GKM3, GKK1, and GKD7 Strains Using in silico Methods

The entire genome of *L. plantarum* GKM3, GKK1, and GKD7 strains was further analyzed using ProbioMinServer to assess the potential risk of probiotics. The analysis revealed no Antibiotic Resistance Genes (ARGs) or Virulence Factors (VFs) in GKM3, GKK1, and GKD7 strains, but two Pathogenic Genes (PGs) were detected (Table 3). While GKD7 contained one plasmid, no plasmids were found in GKK1 and GKM3. Moreover, GKK1 and GKM3 strains each exhibited three prophage regions, while GKD7 had two prophage regions. Overall, the ProbioMinServer-based composition analysis of ARGs, VFs, and PGs resulted in a PPRS of 2.00 for GKM3, GKK1, and GKD7, indicating a low-risk safety profile for these strains.

### Safety Assessment of *L. plantarum* GKM3, GKK1, and GKD7 Strains In Vivo

An acute toxicity test was conducted to evaluate the safety of *L. plantarum* GKM3, GKK1, and GKD7 strains in Sprague-Dawley rats. Male and female rats were given a daily dose of 2000 mg/kg body weight *L. plantarum* GKM3, GKK1, and GKD7 solution for 7 consecutive days. On day 8, all rats survived, and there were no significant differences observed in body weight gain, alanine aminotransferase (ALT), aspartate aminotransferase (AST), and blood urea nitrogen (BUN), except for a significant increase in BUN levels in the female group treated with GKK1 (*p* < 0.05; Table 4). However, upon examination of the liver and kidneys during the post-mortem, no changes related to the treatment were observed. Despite the increase in BUN levels, there was a lack of dose-response, inconsistency across sexes, and absence of associated clinical or pathological findings, suggesting that the treatment was generally safe.

### Effect of *L. plantarum* GKM3, GKK1, and GKD7 Strains on Host Immunity

**Fig. 2:**
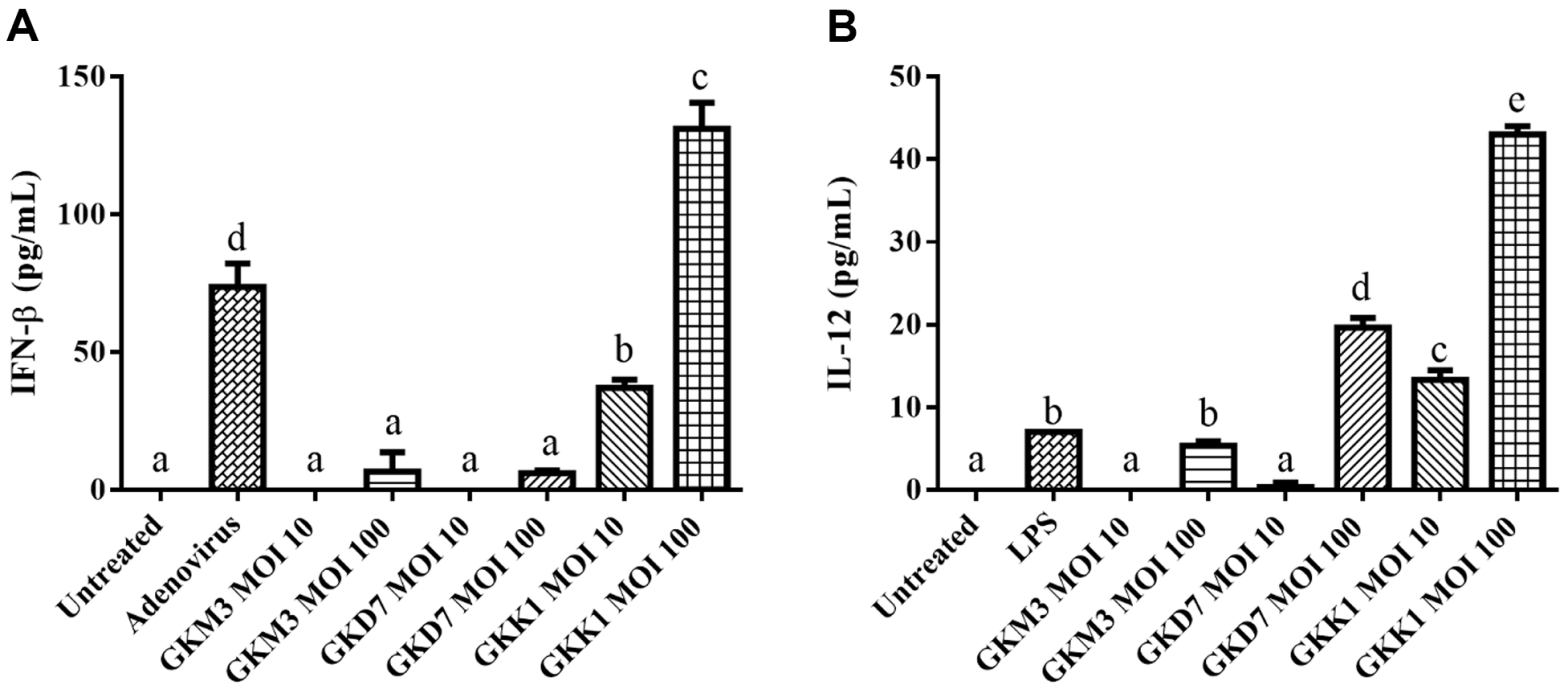
Effects of *L. plantarum* GKM3, GKK1, and GKD7 at MOI values of 10 and 100 bacteria/cell on (A) IFN-β and (B) IL-12 expression in bone marrow-derived dendritic cells from mice. Positive controls using 250 MOI adenovirus and 100 ng/ml LPS were employed to induce the production of IFN-β and IL-12, respectively. The data presented are the means ± standard deviation (*n* = 3). Statistical significance was determined using one-way ANOVA with Tukey's post-test. The letters a, b, and c indicate statistically significant differences (*p* < 0.05).

After conducting the safety assessment, the beneficial effects of GKM3, GKK1, and GKD7 strains on the hosts were evaluated. Bone marrow-derived dendritic cells from mice were either untreated or challenged with *L. plantarum* GKM3, GKK1, and GKD7 at MOI values of 10 and 100 bacteria/cell for a 24-h period. Notably, GKK1 demonstrated a higher level of IFN-β induction compared to GKD7, GKM3, and the control adenovirus, in a dose-dependent manner (*p* < 0.05, Fig. 2A). Additionally, GKK1 also exhibited a significantly higher level of IL-12 induction in a dose-dependent manner, when compared to GKD7, GKM3, and the control LPS (*p* < 0.05, Fig. 2B).

### Identification of Candidate Metabolites Involved in Cytokine Secretion

**Fig. 3:**
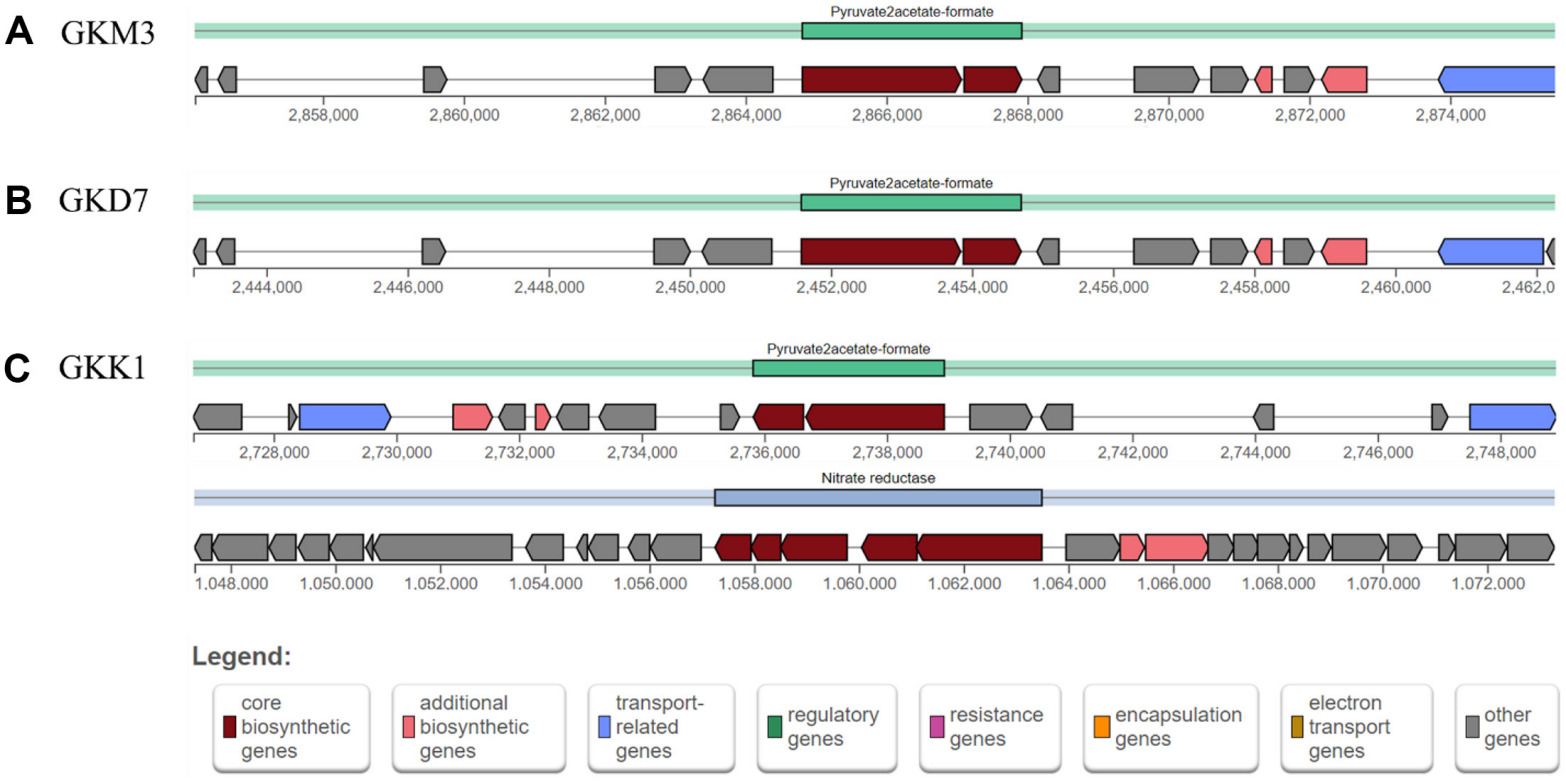
The gutSMASH run was performed for *L. plantarum* strains (A) GKM3, (B) GKD7, and (C) GKK1. All strains have a Pyruvate to acetate-formate metabolic gene cluster type, with GKK1 having an additional nitrate reductase region.

To identify candidate metabolites involved in the secretion of pro-inflammatory cytokines, web tools gutSMASH, antiSMASH, and BAGEL4 were used to detect primary metabolites, secondary metabolites, and bacteriocins, respectively. A summary of predicted compounds, types and similarity scores are shown in Supplementary Tables S1-S3. A metabolic gene cluster "Pyruvate2acetate-formate" responsible for converting pyruvate into acetate and formate was identified in all strains, which is crucial for short-chain fatty acid production (Fig. 3 and Table S1) [46]. Interestingly, only GKK1 strain possesses a nitrate reductase region, which may convert nitrate and nitrite to ammonia and nitric oxide (NO), serving as a signaling component in immune response (Fig. 3A-3C and Table S1).

**Fig. 4:**
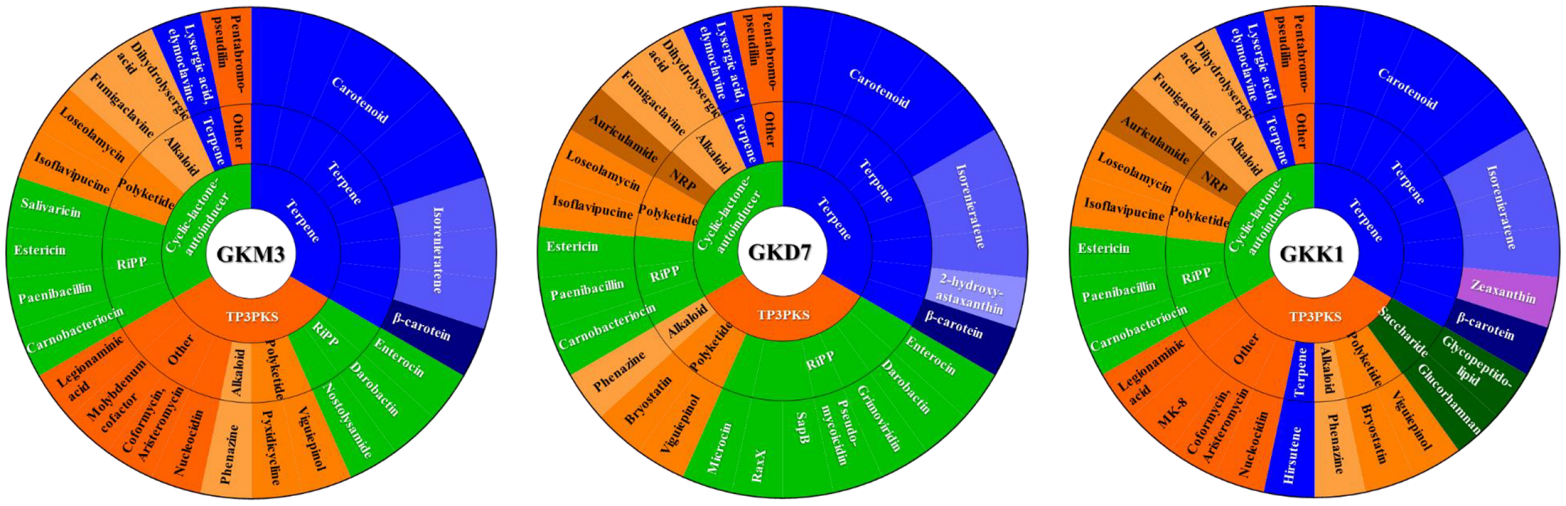
Predicted secondary metabolite biosynthesis gene clusters similar in the MIBig database within *L. plantarum* GKM3, GKK1, and GKD7 strains using antiSMASH with the "strict" setting. RiPP: Ribosomal synthesized and post-translationally modified peptides; T3PKS: type III polyketide synthase; NRP: non-ribosomally produced peptides; MK: menaquinone; and Other: cluster containing a secondary metabolite-related protein that does not fall into any other category.

When analyzing secondary metabolites using antiSMASH with the "strict" strictness, all strains exhibited three metabolite regions: Type III polyketide synthase (T3PKS), terpene, and cyclic-lactone-autoinducer metabolism gene clusters (Fig. 4 and Table S2). These gene clusters were further categorized into different types. GKM3 had a total of 3 alkaloids, 4 polyketides, 7 ribosomally synthesized and post-translationally modified peptides (RiPPs), 11 terpenes, and 5 unidentified compounds. GKD7 had a total of 3 alkaloids, 4 polyketides, 1 nonribosomal peptide (NRP), 10 RiPPs, 11 terpenes, and 1 unidentified compound. GKK1 had a total of 3 alkaloids, 4 polyketides, 1 NRP, 3 RiPPs, 2 saccharides, 12 terpenes, and 5 unidentified compounds. Upon comparison between these strains, all compounds within the cyclic-lactone-autoinducer and terpene regions were shared between GKK1 and GKD7, except for 2-hydroxyastaxanthin and zeaxanthin. Furthermore, compounds within the T3PKS regions were shared between GKM3, GKK1, and GKD7, except for nostolysamide A/B and molybdenum cofactor found in GKM3, grimoviridin, pseudomycoicidin, SapB, RaxX, microcin N found in GKD7, and glycopeptidolipid, glucorhamnan, and menaquinone-8 (MK-8) found in GKK1.

**Fig. 5:**
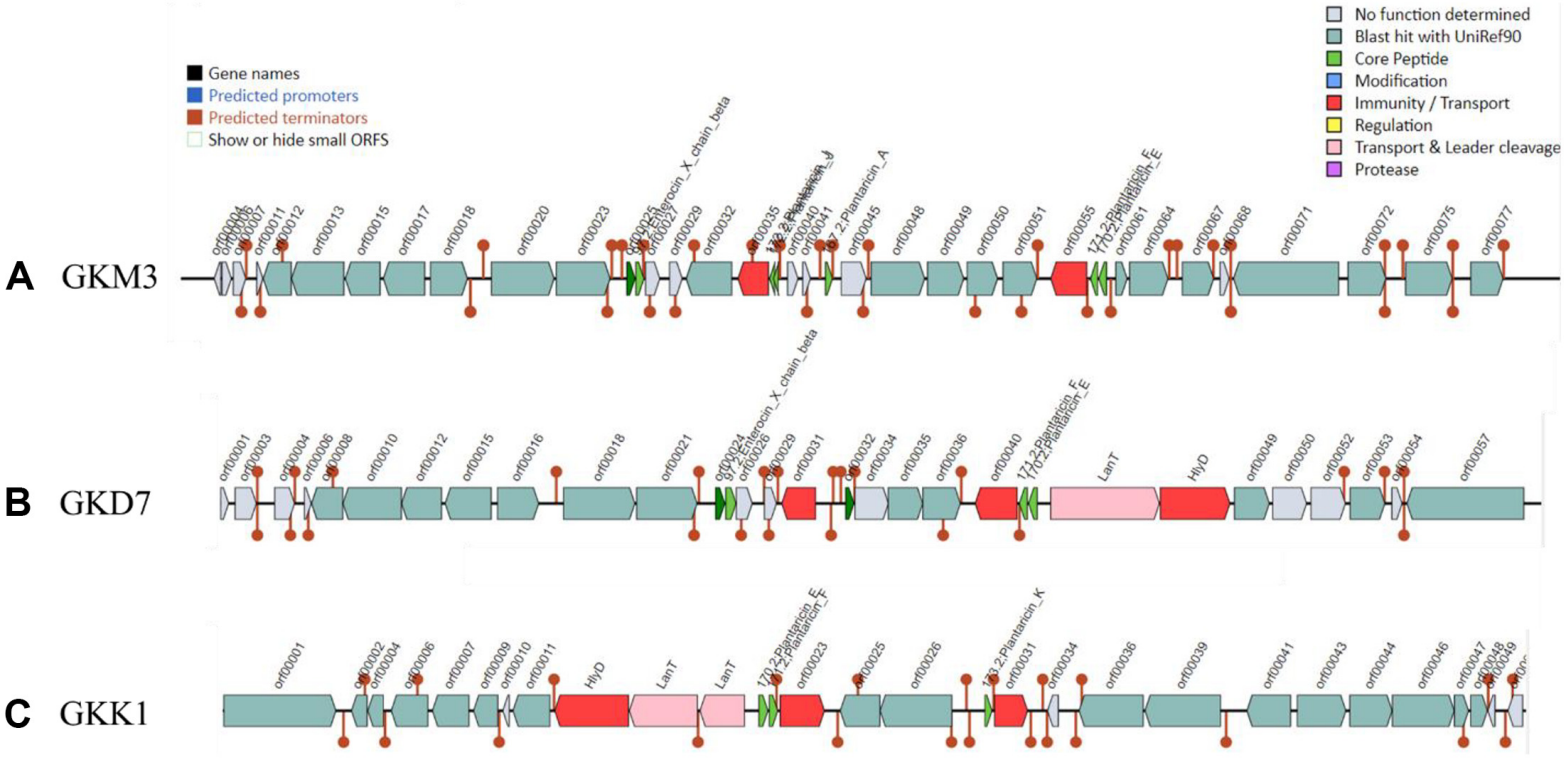
Bacteriocin cluster genes in *L. plantarum* (A) GKM3, (B) GKD7, and (C) GKK1 strains predicted with the BAGEL 4 webserver.

The gene clusters responsible for producing bacteriocins were identified using the BAGEL4 software. All strains contain plantarcins E and F, while only the GKD7 and GKM3 strains contain the enterocin X chain beta (Fig. 5 and Table S3). Furthermore, GKM3 genomes were found to have plantaricins A and J, while GKK1 had plantaricin K.

### Confirmation of Nitrate Reductase Gene among Three Strains

**Fig. 6:**
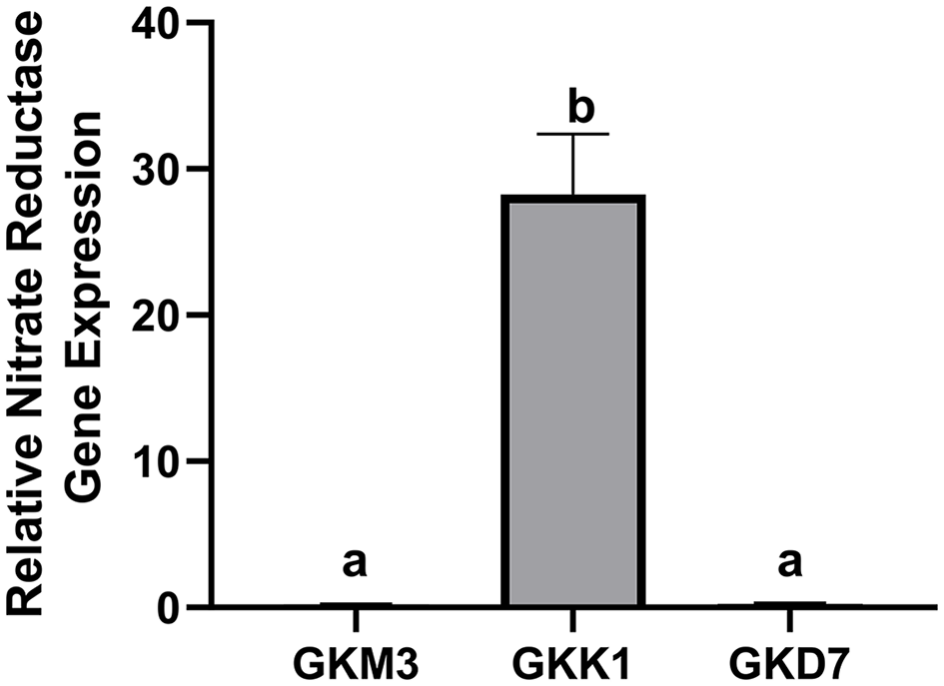
The relative nitrate reductase gene expression normalized to the control gene 16S rRNA across three strains. The data are presented as the mean ± standard deviation (*n* = 3). Statistical significance was determined using oneway ANOVA with Tukey's post-test. The letters a and b indicate statistically significant differences (*p* < 0.05).

To validate whether the GKK1 strain alone has a nitrate reductase region that could potentially convert nitrate and nitrite to ammonia and NO, the expression of the nitrate reductase *nar*G gene was examined via RT-PCR among three strains. The 16S rRNA was used as an internal control. As illustrated in Fig. 6, GKK1 showed a significantly higher expression of nitrate reductase compared to GKD7 and GKM3. These observations align with outcomes expected from the gutSMASH predictions.

### Antiinflammtory Effect of Plantaricin A

**Fig. 7:**
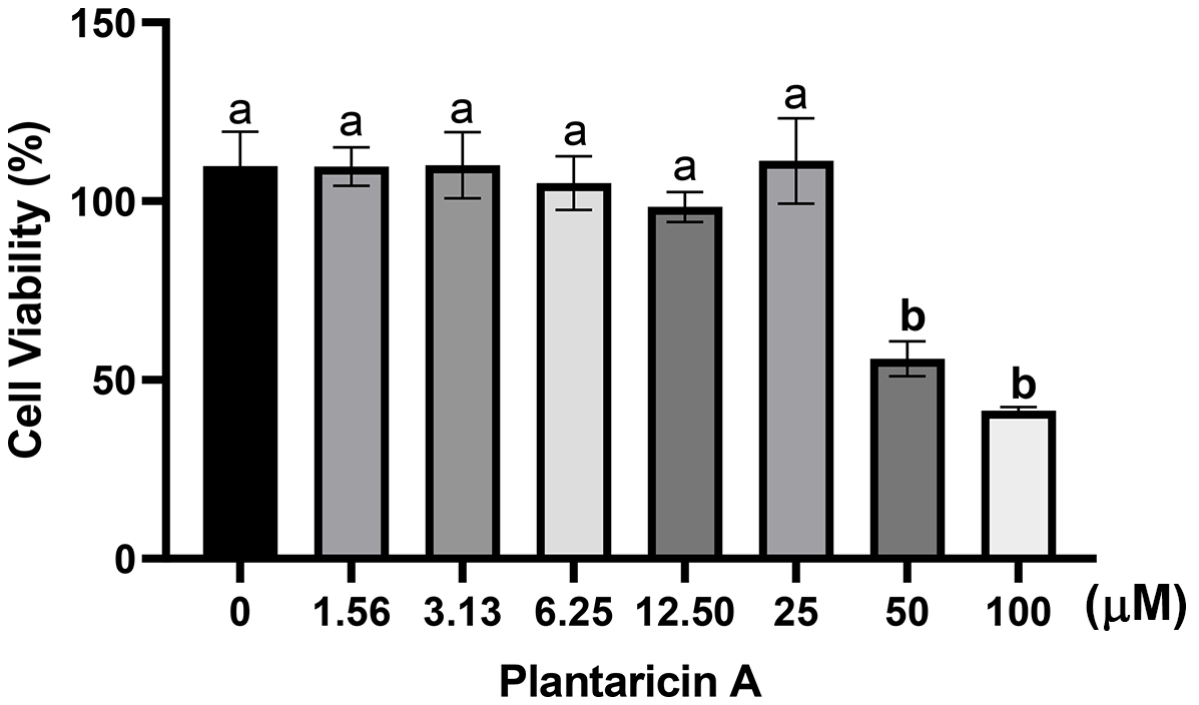
Concentration–effect curves of plantaricin A in RAW 264.7 cells were assessed after 24 h of exposure using the MTT assay. The results were expressed as a percentage of cell viability relative to the untreated controls and represented as the mean ± standard deviation of three independent experiments. Statistical significance was determined using one-way ANOVA with Tukey's post-test. The letters a and b indicate statistically significant differences (*p* < 0.05).

**Fig. 8:**
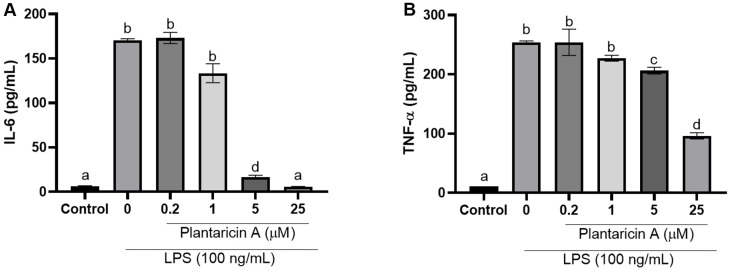
Effects of plantaricin A on LPS-induced (A) IL-6 and (B) TNF-α production. RAW 264.7 cells were pretreated with plantaricin A for1 h before being exposed to 100 ng/ml LPS. Following a 24 h incubation period, IL-6 and TNF- α production was measured by ELISA. The resulting data, presented as means ± SD, was analyzed through one-way ANOVA with Tukey’s multiple comparisons post hoc test. Distinct letters signify statistically significant differences at *p* < 0.05.

Among bacteriocin candidates, plantaricin A is the only one that has been commercialized. For the first time, it was tested in this study for potential anti-inflammatory properties. RAW 264.7 cells were exposed to plantaricin A at concentrations ranging from 1.56–100 μM for 24 h. The observations revealed no cytotoxic effects of plantaricin A up to a concentration of 25 μM (Fig. 7). The anti-inflammatory impact of plantaricin A was further investigated by examining its effects on IL-6 (Fig. 8A) and TNF-α (Fig. 8B) production in LPS-induced RAW 267.4 cells, using ELISA. LPS treatment was observed to significantly increase (*p* < 0.05) the IL-6 and TNF-α levels by 26.8- and 22.4-fold, respectively. However, these LPS-induced increases were dose-dependently reversed by plantaricin A treatments. Results showed that 5–25 μM of plantaricin A significantly reduced the production of IL-6 and TNF-α when compared to LPS-stimulated control cells.

## Discussion

Nowadays, even though the world has moved beyond the shadows of the pandemic, there is still a high demand for immune-related products [47]. This demand stems from peoplés desire to be prepared not only for pandemics but also for small viral outbreaks [47]. Probiotics have been found to have a positive impact on the composition of intestinal microflora and can interact with different immune cells, thereby improving immune functions [48]. As a result, the use of probiotics in various foods has significantly increased [49]. However, since the effectiveness of probiotics depends on the species or strain, they need to possess specific characteristics such as safety, functionality, and beneficial properties. Therefore, this study aimed to evaluate the safety and potential probiotic properties of three newly identified *L. plantarum* strains (GKM3, GKK1, and GKD7) from Taiwan using *in vivo* toxicity tests in combination with whole-genome sequencing.

From a genetic standpoint, all three strains were identified as *L. plantarum* using the ProbioMinServer. These strains each possess a single, circular chromosome ranging from 2.99-3.10 Mbp, containing 2958-3258 coding sequences, and an average GC content of 45% (Table 2), which aligns with earlier studies outlining the genomic structure of various *L. plantarum* strains [50]. Furthermore, our pan-genome analysis, which classifies genes into core, dispensable, and strain-specific categories, aligns closely with an in-depth analysis of 108 full *L. plantarum* genomes from the NCBI GenBank database [51]. Among these three strains, they share 2139 core genes, 689 dispensable genes, and 1590 strain-specific genes, indicating that these *L. plantarum* strains possess essential probiotic characteristics while also exhibiting some unique traits.

To assess the safety of these three *L. plantarum* strains, the PPRS (classified as low-risk (≤4), medium-risk (4-6), and high-risk (≥6)) was evaluated by analyzing ARGs, VFs, PGs, plasmids, and prophages. While the PPRS is not a universally adopted tool, it is recognized and used within the field of microbiology and probiotic research [52]. The reliability of the PPRS stems from its inclusion of different elements that can contribute to the potential risk of a probiotic strain. Through this comprehensive approach, it offers a more complete picture of a strain's safety profile compared to methods that only consider a single factor. All three strains have a PPRS of 2.00, indicating a low-risk safety profile for these strains [27]. Furthermore, acute oral toxicity tests were conducted to confirm the safety of these strains for human consumption. Oral toxicity tests using rats can provide evidence for the safety of human consumption due to the similarity in the physiology of rats and humans [53]. In oral toxicity test, none of the *L. plantarum* strains caused adverse events in the rats. Despite a significant change in BUN was observed in the female GKK1 group, the lack of dose-response, inconsistency across sexes, and absence of associated clinical or pathological findings suggested that this difference could be considered as normal biological variations. Taken together, these results demonstrate that a single dose of 2000 mg/kg/day (9.7 × 10^12^ CFU/day in human dosages assuming a body mass of 60 kg) of *L. plantarum* GKM3, GKK1, and GKD7 strains can be considered safe as probiotic strains. Nevertheless, it is important to note that this study did not carry out several essential tests such as hematology measurements, long-term toxicity, genotoxicity, and teratogenicity evaluations. These are crucial for medical applications and their absence may limit the understanding of potential risks associated with these strains.

To evaluate the health benefits of *L. plantarum* GKM3, GKK1, and GKD7, the secretion of TNF-β and IL-12 by dendritic cells was measured after stimulation with these strains. Among the three strains in this study, activation of dendritic cells by GKK1 resulted in the most significant enhancement of both IFN-β and IL-12 production. Activation of dendritic cells by viruses primarily induces IFN-β, but certain bacteria have also been shown to be potent inducers of IFN-β [54]. IFN-β, in turn, up-regulates a high number of viral defense genes and stimulates the production of the Th1 inducing cytokine IL-12 through ligation of the specific IFN type I receptor, IFNAR [55]. Hence, enhancing IFN-β induction by bacterial activation may represent an important means to increase antiviral defense and cellular immunity. Additionally, IL-12 plays key roles in inducing a Th1 effector immune response and enhancing cellular immunity [56]. Studies have demonstrated that mice deficient in IL-12 are more susceptible to various pathogens, including *Leishmania*, *Plasmodium*, *Toxoplasma*, *Cryptococcus*, *Francisella*, and *Mycobacteria* [56]. Consequently, IL-12 is a critical factor in the selection of immunostimulatory probiotic strains.

Web tools gutSMASH, antiSMASH, and BAGEL4 were utilized to perform an in-depth analysis to identify potential primary metabolites, secondary metabolites, and bacteriocins that could be responsible for the secretion of pro-inflammatory cytokines. Regarding primary metabolites, it was found that all strains possess pyruvate formate-lyase gene clusters (pyruvate to acetate/formate), which is essential for short-chain fatty acid production. Notably, among the strains studied, only the GKK1 strain exhibited the presence of a nitrate reductase region. This region potentially enables the conversion of nitrate and nitrite to ammonia and nitric oxide (NO) within the gut environment [57]. Previous studies have demonstrated that when *L. plantarum* is grown in the presence of millimolar levels of nitrate at low oxygen concentrations (4% or lower), it has the capacity to produce ammonia through nitrate reduction [57]. Moreover, the abundant production of lactic acid by *L. plantarum* creates an acidic environment, which further facilitates the conversion of nitrite to NO [57].

While NO is primarily recognized for its antibacterial properties, it also acts as a critical effector molecule involved in immune regulation and host defense [58]. Interestingly, the concentration of NO has been shown to exert different effects on immune cells [59, 60]. At high concentrations, nitric oxide has been found to promote Th2 differentiation by suppressing IL-12 synthesis [59]. Conversely, at low concentrations, NO can stimulate T cells to express IL-12 and promote Th1 differentiation [60]. Furthermore, recent research has indicated that the administration of low-dose nitric oxide donors can elevate the levels of splenic cytokines such as interferon-gamma (IFN-γ) and TNF-α, while simultaneously reducing the levels of IL-6 and IL-10, suggesting a shift towards Th1 cell responses [61]. Based on these intriguing findings, it is plausible to hypothesize that the presence of the nitrate reductase region in the GKK1 strain may contribute to the enhancement of both IFN-β and IL-12 production through the production of NO. To confirm if the GKK1 strain alone possesses a nitrate reductase region capable of converting nitrate and nitrite to ammonia and NO, we examined the expression of the nitrate reductase *nar*G gene in three strains via RT-PCR, using the 16S rRNA as an internal control. As depicted in Fig. 6, GKK1 exhibited significantly higher expression of nitrate reductase compared to GKD7 and GKM3, which aligned with the gutSMASH predictions.

The investigation of secondary metabolites is complex because their production is not universal under all conditions [62]. As a result, their actual functions remain elusive and require further exploration. In this study, examination of several gene clusters responsible for secondary metabolite production using antiSMASH revealed a shared presence among GKM3, GKD7, and GKK1, with the exception of nostolysamide A/B and molybdenum cofactor found in GKM3, grimoviridin, pseudomycoicidin, SapB, RaxX, microcin N found in GKD7, and glycopeptidolipid, glucorhamnan, and menaquinone-8 (MK-8) found exclusively in GKK1. While the immune function of most of these secondary metabolites remains unknown, it is worth noting that glucorhamnan has been scientifically reported to enhance immune responses. Previous studies have demonstrated that *Ruminococcus gnavus* produces a proinflammatory polysaccharide called glucorhamnan, which potently induces the secretion of TNF-α by dendritic cells through Toll-like receptor 4 [63]. Considering that the GKK1 strain harbors the gene clusters responsible for glucorhamnan production, while GKM3 and GKD7 do not, it is plausible to hypothesize that glucorhamnan may contribute to the production of IFN-β and IL-12. Nevertheless, it is essential to conduct further studies to validate and expand upon this assumption.

Bacteriocins are protein compounds produced by bacteria that have antimicrobial properties against specific pathogens [64]. Recent studies have shown that bacteriocins cannot only act as a defense mechanism for bacteria, but they can also cause changes in the gut population by affecting the immune system response [48]. In order to gain further insights, the genes responsible for bacteriocin biosynthesis in the strains GKM3, GKD7, and GKK1 were thoroughly analyzed using BAGEL4. The results revealed that all strains contain plantarcins E/F, while only GKD7 and GKM3 strains contain the enterocin X chain beta. Additionally, plantaricins A and J were found in the genome of GKM3, while GKK1 had plantaricin K. Previous research has suggested that plantaricin A functions as a peptide pheromone that induces bacteriocin production, while plantarcins E/F, J/K, and enterocin Xα/β are two-peptide plantaricins with notable antimicrobial properties [65, 66]. It is worth noting that among the identified bacteriocins, only plantarcins E/F have been scientifically confirmed to induce IL-10 production both in vitro [67] and in vivo [68].

For the first time, it is been demonstrated that commercial plantaricin A can significantly reduce LPS-induced inflammation. Existing research [69] reveals that the plantaricin A gene-encoded 48-residue precursor "MKIQIKGMKQLSNKEMQKIVGGKSSAYSLQMGATAIKQVKKLFKKWGW" results in three variants of plantaricin A. These variants, all originating from the same precursor, include a 26-residue full-length peptide (PlnA-26) "KSSAYSLQMGATAIKQVKKLFKKWGW", along with two N-terminally truncated forms with 23 (PlnA-23) "AYSLQMGATAIKQVKKLFKKWGW" and 22 (PlnA-22) residues "YSLQMGATAIKQVKKLFKKWGW". Importantly, these variants have antimicrobial and pheromone activities [70]. The commercial plantaricin A that was purchased included the sequence "AYSLQMGATAIKQVKKLFKKWGW", which is an exact match to the gene encoded for GKM3 as per BAGEL4. This key discovery points to the potential therapeutic use of not just PlnA-23, but also PlnA-26 and PlnA-22 in treating inflammatory conditions, which calls for further exploration.

It is noteworthy that plantaricin A production was also observed in the DSM 20174 type strain, as confirmed by the BAGEL4 tool (Fig. S1). Earlier studies have also hinted at the anti-inflammatory effects of this type strain [71]. These findings suggest that plantaricin A, found in both GKM3 and DSM 20174, could play a significant role in providing an anti-inflammatory effect. It is important to note, however, that GKM3, GKD7 and DSM 20174 also contain additional bacteriocins. For future studies, looking into the immune functions of these bacteriocins may provide more insights into inflammation control and open new possibilities for therapeutic interventions.

## Conclusion

In conclusion, the newly identified *L. plantarum* strains (GKM3, GKK1, and GKD7) demonstrated a safe profile and showed potential for immune enhancement. Safety evaluations confirmed their suitability for human consumption, and GKK1 exhibited the most significant enhancement of immune-stimulating cytokine production. Bioinformatics analysis highlighted potential metabolic pathways and secondary metabolites associated with immune modulation, including the presence of a nitrate reductase region in GKK1 and glucorhamnan-related gene clusters. Nitrate reductase expression was confirmed using qPCR and was highest in GKK1. Furthermore, this study presented the anti-inflammatory effects of plantaricin A for the first time. Overall, these findings point to the potential benefits of using these strains in immune-related applications.

## Supplemental Materials

Supplementary data for this paper are available on-line only at http://jmb.or.kr.



## Figures and Tables

**Table 1:** Primers used in this study.

Target Gene	Sequence 5'-3'	Annealing temperature	Size (bp)
*nar*G	F: TCGCCSATYCCGGCSATGTC R: GAGTTGTACCAGTCRGCSGAYTCSG	58	173
16S rRNA	F: CCTACGGGAGGCAGCAGTAG R: CAACAGAGCTTTACGATCCGAAA	52	101

**Table 2:** General genome features of *L. plantarum* strains GKM3, GKK1, and GKD7.

Sample	Source	Size (Mbp)	GC (%)	CDS	rRNA	tRNA
GKD7	Taiwanese Kimchi	3.09	44.52	3258	16	68
GKK1	Pickled Chili	3.10	44.58	3271	16	63
GKM3	Pickled Mustard Green	2.99	44.64	2958	16	67

**Table 3:** Safety analysis of *L. plantarum* GKM3, GKK1, and GKD7 strains.

Strains	GKK1	GKD7	GKM3
Antibiotic Resistance Genes (ARGs)			
Comprehensive Antibiotic Resistance Database (CARD) v4.0.0	0	0	0
Virulence factors (VFs)			
Virulence Factor Database	0	0	0
Pathogenic genes (PGs)			
Pathogen Host Interactions Database v4.14	2	2	2
Plasmid			
PlasmidFinder v2.0.1	0	1	0
Prophage			
Phigaro v2.3.0	3	2	3
Probiotic Potential Risk Score	2	2	2
Probiotic Potential Risk Score (PPRS) = √ *N_ARG_* ^2^ + *N_VF_* ^2^ +*N_ARG_* ^2^			
Probiotic Potential Risk Score is classified as low-risk (≤4), medium-risk (4-6), and high-risk (≥6)			

**Table 4:** Acute oral toxicity study of *L. plantarum* GKM3, GKK1, and GKD7 strains.

Parameters				
Male	Control	GKM3	GKK1	GKD7
Weight change (g)	29.1 ± 4.4	32.9 ± 6.0	28.3 ± 6.0	31.0 ± 4.8
ALT (U/L)	66.6 ± 12.5	67.2 ± 20.2	55.6 ± 6.3	93.0 ± 38.6
AST (U/L)	135.2 ± 29.8	134.4 ± 35.8	126.6 ± 33.7	142.6 ± 59.2
BUN (mg/dL)	19.9 ± 2.9	19.9 ± 2.2	18.8 ± 3.6	21.9 ± 2.8
Female	Control	GKM3	GKK1	GKD7
Weight change (g)	6.0 ± 1.6	8.1 ± 2.5	6.7 ± 2.9	6.2 ± 4.2
ALT (U/L)	50.4 ± 6.6	46.8 ± 3.11	41.2 ± 5.2	55.6 ± 10.7
AST (U/L)	93.8 ± 14.8	89.0 ± 3.67	90.4 ± 6.4	102 ± 28
BUN (mg/dL)	21.1 ± 2.8	19.92 ± 2.3	45.4 ± 15.4*	26.0 ± 5.9

Results are expressed as mean ± SD (*n* = 5) and analyzed by One-way ANOVA followed by Duncan's multiple range test

*Significantly different from the control at *p* < 0.05

## References

[1] Sender R, Fuchs S, Milo R (2016). Are we really vastly outnumbered? Revisiting the ratio of bacterial to host cells in humans. Cell.

[2] Lynch JB, Hsiao EY (2019). Microbiomes as sources of emergent host phenotypes. Science.

[3] Olvera-Rosales LB, Cruz-Guerrero AE, Ramírez-Moreno E, Quintero-Lira A, Contreras-López E, Jaimez-Ordaz J (2021). Impact of the gut microbiota balance on the health-disease relationship: The importance of consuming probiotics and prebiotics. Foods.

[4] Hou K, Wu Z-X, Chen X-Y, Wang J-Q, Zhang D, Xiao C (2022). Microbiota in health and diseases. Signal Transduct. Target. Ther..

[5] Tsai YL, Lin TL, Chang CJ, Wu TR, Lai WF, Lu CC (2019). Probiotics, prebiotics and amelioration of diseases. J. Biomed. Sci..

[6] Mack DR (2005). Probiotics-mixed messages. Can. Fam. Physician.

[7] Kho ZY, Lal SK (2018). The human gut microbiome - A potential controller of wellness and disease. Front. Microbiol..

[8] Cortes-Perez NG, de Moreno de LeBlanc A, Gomez-Gutierrez JG, LeBlanc JG, Bermúdez-Humarán LG (2021). Probiotics and trained immunity. Biomolecules.

[9] Netea Mihai G, Quintin J, van der Meer Jos WM (2011). Trained immunity: A memory for innate host defense. Cell Host Microbe.

[10] McFarland LV, Evans CT, Goldstein EJC (2018). Strain-specificity and disease-specificity of probiotic efficacy: a systematic review and meta-analysis. Front. Med..

[11] Wick RR, Judd LM, Holt KE (2019). Performance of neural network basecalling tools for Oxford nanopore sequencing. Genome Biol..

[12] De Coster W, D'Hert S, Schultz DT, Cruts M, Van Broeckhoven C (2018). NanoPack: visualizing and processing long-read sequencing data. Bioinformatics.

[13] Kolmogorov M, Yuan J, Lin Y, Pevzner PA (2019). Assembly of long, error-prone reads using repeat graphs. Nat. Biotechnol..

[14] Vaser R, Sović I, Nagarajan N, Šikić M (2017). Fast and accurate de novo genome assembly from long uncorrected reads. Genome Res..

[15] Li H (2018). Minimap2: pairwise alignment for nucleotide sequences. Bioinformatics.

[16] Ltd. ONT (2021). Medaka.

[17] Huang YT, Liu PY, Shih PW (2021). Homopolish: a method for the removal of systematic errors in nanopore sequencing by homologous polishing. Genome Biol..

[18] Gurevich A, Saveliev V, Vyahhi N, Tesler G (2013). QUAST: quality assessment tool for genome assemblies. Bioinformatics.

[19] Simão FA, Waterhouse RM, Ioannidis P, Kriventseva EV, Zdobnov EM (2015). BUSCO: assessing genome assembly and annotation completeness with single-copy orthologs. Bioinformatics.

[20] Richter M, Rosselló-Móra R, Oliver Glöckner F, Peplies J (2016). JSpeciesWS: a web server for prokaryotic species circumscription based on pairwise genome comparison. Bioinformatics.

[21] Kwon YJ, Chun BH, Jung HS, Chu J, Joung H, Park SY (2021). Safety assessment of *Lactiplantibacillus* (formerly *Lactobacillus* ) plantarum Q180. J. Microbiol. Biotechnol..

[22] Dereeper A, Summo M, Meyer DF (2022). PanExplorer: a web-based tool for exploratory analysis and visualization of bacterial pangenomes. Bioinformatics.

[23] Zhao Y, Wu J, Yang J, Sun S, Xiao J, Yu J (2012). PGAP: pan-genomes analysis pipeline. Bioinformatics.

[24] Page AJ, Cummins CA, Hunt M, Wong VK, Reuter S, Holden MT (2015). Roary: rapid large-scale prokaryote pan genome analysis. Bioinformatics.

[25] Perrin A, Rocha EPC (2021). PanACoTA: a modular tool for massive microbial comparative genomics. NAR Genom. Bioinform..

[26] Tatusov RL, Galperin MY, Natale DA, Koonin EV (2000). The COG database: a tool for genome-scale analysis of protein functions and evolution. Nucleic Acids Res..

[27] Bai Z, Zhang N, Jin Y, Chen L, Mao Y, Sun L (2022). Comprehensive analysis of 84 *Faecalibacterium prausnitzii* strains uncovers their genetic diversity, functional characteristics, and potential risks. Front. Cell. Infect. Microbiol..

[28] Liu YY, Hsu CY, Yang YC, Huang CH, Chen CC (2023). ProbioMinServer: an integrated platform for assessing the safety and functional properties of potential probiotic strains. Bioinform. Adv..

[29] Rychen G, Aquilina G, Azimonti G, Bampidis V, Bastos ML, Bories G (2018). Guidance on the characterisation of microorganisms used as feed additives or as production organisms. EFSA J..

[30] McArthur AG, Waglechner N, Nizam F, Yan A, Azad MA, Baylay AJ (2013). The comprehensive antibiotic resistance database. Antimicrob. Agents Chemother..

[31] Bortolaia V, Kaas RS, Ruppe E, Roberts MC, Schwarz S, Cattoir V (2020). ResFinder 4.0 for predictions of phenotypes from genotypes. J. Antimicrob. Chemother..

[32] Feldgarden M, Brover V, Gonzalez-Escalona N, Frye JG, Haendiges J, Haft DH (2021). AMRFinderPlus and the reference gene catalog facilitate examination of the genomic links among antimicrobial resistance, stress response, and virulence. Sci. Rep..

[33] Chen L, Yang J, Yu J, Yao Z, Sun L, Shen Y (2005). VFDB: a reference database for bacterial virulence factors. Nucleic Acids Res..

[34] Malberg Tetzschner AM, Johnson JR, Johnston BD, Lund O, Scheutz F (2020). In silico genotyping of *Escherichia coli* isolates for extraintestinal virulence genes by use of whole-genome sequencing data. J. Clin. Microbiol..

[35] OECD (2002). Test No. 423: Acute Oral toxicity - Acute Toxic Class Method, Ed.

[36] Administration UFaD (2005). Guidance for Industry: Estimating the Maximum Safe Starting Dose in Initial Clinical Trials for Therapeutics in Adult Healthy Volunteers.

[37] Lee YL, Hsu LH, Kuo YH, Lee CC (2019). Caffeic amide derivatives inhibit allergen-induced bone marrow-derived dendritic cell maturation. Pharmacol. Rep..

[38] Juan SH, Lee TS, Tseng KW, Liou JY, Shyue SK, Wu KK (2001). Adenovirus-mediated heme oxygenase-1 gene transfer inhibits the development of atherosclerosis in apolipoprotein E-deficient mice. Circulation.

[39] van Heel AJ, de Jong A, Song C, Viel JH, Kok J, Kuipers OP (2018). BAGEL4: a user-friendly web server to thoroughly mine RiPPs and bacteriocins. Nucleic Acids Res..

[40] Pascal Andreu V, Roel-Touris J, Dodd D, Fischbach Michael A, Medema Marnix H (2021). The gutSMASH web server: automated identification of primary metabolic gene clusters from the gut microbiota. Nucleic Acids Res..

[41] Medema MH, Blin K, Cimermancic P, de Jager V, Zakrzewski P, Fischbach MA (2011). antiSMASH: rapid identification, annotation and analysis of secondary metabolite biosynthesis gene clusters in bacterial and fungal genome sequences. Nucleic Acids Res..

[42] Bru D, Sarr A, Philippot L (2007). Relative abundances of proteobacterial membrane-bound and periplasmic nitrate reductases in selected environments. Appl. Environ. Microbiol..

[43] Wasfi R, Abd El-Rahman OA, Zafer MM, Ashour HM (2018). Probiotic *Lactobacillus* sp. inhibit growth, biofilm formation and gene expression of caries-inducing *Streptococcus mutans*. J. Cell. Mol. Med..

[44] Ma Y, Zilles JL, Kent AD (2019). An evaluation of primers for detecting denitrifiers via their functional genes. Environ. Microbiol..

[45] van Meerloo J, Kaspers GJ, Cloos J (2011). Cell sensitivity assays: the MTT assay. Methods Mol. Biol..

[46] Koh A, De Vadder F, Kovatcheva-Datchary P, Bäckhed F (2016). From dietary fiber to host physiology: Short-chain fatty acids as key bacterial metabolites. Cell.

[47] Hamulka J, Jeruszka-Bielak M, Górnicka M, Drywień ME, Zielinska-Pukos MA (2020). Dietary supplements during COVID-19 outbreak.results of google trends analysis supported by PLifeCOVID-19 online studies. Nutrients.

[48] Umair M, Jabbar S, Zhaoxin L, Jianhao Z, Abid M, Khan KR (2022). Probiotic-based bacteriocin: Immunity supplementation against viruses. An updated review. Front. Microbiol..

[49] Vera-Santander VE, Hernández-Figueroa RH, Jiménez-Munguía MT, Mani-López E, López-Malo A (2023). Health benefits of consuming foods with bacterial probiotics, postbiotics, and their metabolites: A review. Molecules.

[50] Zhang W, Ji H, Zhang D, Liu H, Wang S, Wang J (2018). Complete genome sequencing of *Lactobacillus plantarum* ZLP001, a potential probiotic that enhances intestinal epithelial barrier function and defense against pathogens in pigs. Front. Physiol..

[51] Mao B, Yin R, Li X, Cui S, Zhang H, Zhao J (2021). Comparative genomic analysis of *Lactiplantibacillus plantarum* isolated from different niches. Genes.

[52] Ma N, Sun J, Li S, Shao M, Ying N, Liu W (2023). A potential risk comprehensive evaluation model of probiotic species based on complete genome sequences. Food Anal. Methods.

[53] Mukherjee P, Roy S, Ghosh D, Nandi SK (2022). Role of animal models in biomedical research: a review. Lab. Anim. Res..

[54] Ali S, Mann-Nüttel R, Schulze A, Richter L, Alferink J, Scheu S (2019). Sources of type I interferons in infectious immunity: Plasmacytoid dendritic cells not always in the driver's seat. Front. Immunol..

[55] Boxx GM, Cheng G (2016). The roles of type I interferon in bacterial infection. Cell Host Microbe.

[56] Tait Wojno ED, Hunter CA, Stumhofer JS (2019). The immunobiology of the interleukin-12 family: room for discovery. Immunity.

[57] Tiso M, Schechter AN (2015). Nitrate reduction to nitrite, nitric oxide and ammonia by gut bacteria under physiological conditions. PLoS One.

[58] Radi R (2018). Oxygen radicals, nitric oxide, and peroxynitrite: Redox pathways in molecular medicine. Proc. Natl. Acad. Sci. USA.

[59] Huang FP, Niedbala W, Wei XQ, Xu D, Feng GJ, Robinson JH (1998). Nitric oxide regulates Th1 cell development through the inhibition of IL-12 synthesis by macrophages. Eur. J. Immunol..

[60] Niedbala W, Wei XQ, Campbell C, Thomson D, Komai-Koma M, Liew FY (2002). Nitric oxide preferentially induces type 1 T cell differentiation by selectively up-regulating IL-12 receptor beta 2 expression via cGMP. Proc. Natl. Acad. Sci. USA.

[61] Li CY, Anuraga G, Chang CP, Weng TY, Hsu HP, Ta HDK (2023). Repurposing nitric oxide donating drugs in cancer therapy through immune modulation. J. Exp. Clin. Cancer Res..

[62] Reshi ZA, Ahmad W, Lukatkin AS, Javed SB (2023). From nature to lab: A review of secondary metabolite biosynthetic pathways, environmental influences, and in vitro approaches. Metabolites.

[63] Henke MT, Kenny DJ, Cassilly CD, Vlamakis H, Xavier RJ, Clardy J (2019). *Ruminococcus gnavus*, a member of the human gut microbiome associated with Crohn's disease, produces an inflammatory polysaccharide. Proc. Natl. Acad. Sci. USA.

[64] Belguesmia Y, Bendjeddou K, Kempf I, Boukherroub R, Drider D (2020). Heterologous biosynthesis of five new class II bacteriocins from *Lactobacillus paracasei* CNCM I-5369 with antagonistic activity against pathogenic *Escherichia coli* strains. Front. Microbiol..

[65] Syaputri Y, Iwahashi H (2020). Characteristics of heterologous plantaricin from *Lactobacillus plantarum* and its future in food preservation. Rev. Agric. Sci..

[66] Hu CB, Malaphan W, Zendo T, Nakayama J, Sonomoto K (2010). Enterocin X, a novel two-peptide bacteriocin from Enterococcus faecium KU-B5, has an antibacterial spectrum entirely different from those of its component peptides. Appl. Environ. Microbiol..

[67] Meijerink M, van Hemert S, Taverne N, Wels M, de Vos P, Bron PA (2010). Identification of genetic loci in *Lactobacillus plantarum* that modulate the immune response of dendritic cells using comparative genome hybridization. PLoS One.

[68] Yin X, Heeney D, Srisengfa Y, Golomb B, Griffey S, Marco M (2018). Bacteriocin biosynthesis contributes to the anti-inflammatory capacities of probiotic *Lactobacillus plantarum*. Benef. Microbes.

[69] Kristiansen PE, Fimland G, Mantzilas D, Nissen-Meyer J (2005). Structure and mode of action of the membrane-permeabilizing antimicrobial peptide pheromone plantaricin A. J Biol. Chem..

[70] Anderssen EL, Diep DB, Nes IF, Eijsink VG, Nissen-Meyer J (1998). Antagonistic activity of *Lactobacillus plantarum* C11: two new two-peptide bacteriocins, plantaricins EF and JK, and the induction factor plantaricin A. Appl. Environ. Microbiol..

[71] Lee HK, Choi SH, Lee CR, Lee SH, Park MR, Kim Y (2015). Screening and characterization of lactic acid bacteria strains with anti-inflammatory activities through in vitro and caenorhabditis elegans model testing. Korean J. Food Sci. Anim. Resour..

