# Supplementary material for: Whole-Genome Sequencing of Three Lactiplantibacillus plantarum Strains Reveals Potential Metabolites for Boosting Host Immunity Safely
Source: J Microbiol Biotechnol. 2024 Jul 31;34(10):2079–90. doi: 10.4014/jmb.2402.02013 (PMC11540610; doi:10.4014/jmb.2402.02013)
Supplement: Supplementary file 1 [file jmb-34-10-2079-supple.pdf]

J. Microbiol. Biotechnol. 2024. 34(10): 2079-2090
https://doi.org/10.4014/jmb.2402.02013

Supplementary Figure and Tables

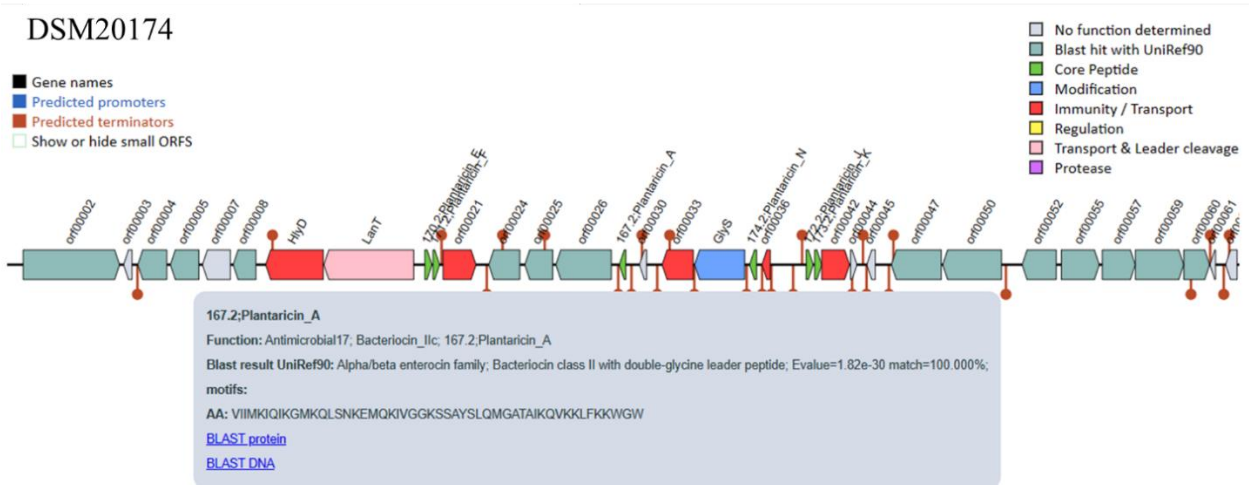

Fig. S1. Predicted bacteriocin cluster genes in *L. plantarum* type strain DSM20174 using the BAGEL 4 webserver.

Table S1. **Summary of primary metabolite gene clusters found in *L. plantarum* GKM3, GKK1, and GKD7.**

Strain	Type	Class	Similarity
GKM3	Pyruvate to acetate-formate	Short chain fatty acid	100%
GKD7	Pyruvate to acetate-formate	Short chain fatty acid	100%
GKK1	Pyruvate to acetate-formate	Short chain fatty acid	100%
	Nitrate reductase	Energy-capturing-related gene clusters	40%

Table S2. Summary of secondary metabolite gene clusters found in *L. plantarum* GKM3, GKK1, and GKD7.

Reference	Type		Compound(s)	Organism	Similarity score
			**GKM3**		
BGC0002674	T3PKS	RiPP	enterocin F4-9	*Enterococcus faecalis*	0.23
BGC0000286	T3PKS	Polyketide	viguiepinol	*Streptomyces sp. KO-3988*	0.23
BGC0001387	T3PKS	Other	nucleocidin	*Streptomyces calvus*	0.22
BGC0002561	T3PKS	Alkaloid	phenazine SA, phenazine SB, phenazine SC	*Streptomyces sp.*	0.19
BGC0002435	T3PKS	Other	coformycin, aristeromycin	*Micromonospora haikouensis*	0.18
BGC0000916	T3PKS	Other	molybdenum cofactor	*Staphylococcus carnosus*	0.17
BGC0001651	T3PKS	Other	legionaminic acid	*Tannerella forsythia 92A2*	0.17
BGC0001940	T3PKS	Polyketide	pyxidicycline A, pyxidicycline B	*Pyxidicoccus fallax*	0.17
BGC0002116	T3PKS	RiPP	darobactin A	*Photorhabdus khanii HGB 1456*	0.17
BGC0002699	T3PKS	RiPP	nostolysamide A, nostolysamide B	*Nostoc punctiforme PCC 73102*	0.16
BGC0000647	terpene	Terpene	carotenoid	*Rhodobacter sphaeroides*	0.45
BGC0000650	terpene	Terpene	carotenoid	*Algoriphagus sp. KK10202C*	0.32
BGC0000645	terpene	Terpene	carotenoid	*Halobacillus halophilus DSM 2266*	0.25
BGC0000648	terpene	Terpene	carotenoid	*Myxococcus xanthus*	0.23
BGC0001456	terpene	Terpene	isorenieratene	*Streptomyces argillaceus*	0.21
BGC0001227	terpene	Terpene	isorenieratene	*Streptomyces collinus Tu 365*	0.21
BGC0000633	terpene	Terpene	carotenoid	*Streptomyces avermitilis*	0.21

BGC0000664	terpene	Terpene	isorenieratene	*Streptomyces griseus subsp. griseus NBRC 13350*	0.2
BGC0000646	terpene	Terpene	β-carotein	*uncultured bacterium*	0.19
BGC0000640	terpene	Terpene	carotenoid	*Enterobacteriaceae bacterium DC404*	0.19
BGC0002579	cyclic-lactone-autoinducer	RiPP	carnobacteriocin XY	*Carnobacterium maltaromaticum*	0.15
BGC0000540	cyclic-lactone-autoinducer	RiPP	paenibacillin	*Paenibacillus polymyxa OSY-DF*	0.12
BGC0000811	cyclic-lactone-autoinducer	Alkaloid	fumigaclavine C	*Aspergillus fumigatus Af293*	0.1
BGC0002667	cyclic-lactone-autoinducer	RiPP	estericin A	*Clostridium esterthеticum*	0.1
BGC0001573	cyclic-lactone-autoinducer	Alkaloid	dihydrolysergic acid	*Claviceps africana*	0.1
BGC0001122	cyclic-lactone-autoinducer	NRP, Polyketide	dihydroisoflavipucine, isoflavipucine	*Aspergillus terreus NIH2624*	0.09
BGC0001267	cyclic-lactone-autoinducer	Terpene	lysergic acid, elymoclavine	*Claviceps fusiformis*	0.09
BGC0000891	cyclic-lactone-autoinducer	Other (Aminocoumarin)	pentabromopseudilin	*Pseudoalteromonas luteoviolacea 2ta16*	0.09
BGC0000624	cyclic-lactone-autoinducer	RiPP	salivaricin CRL1328 α peptide, salivaricin CRL1328 β peptide	*Lactobacillus salivarius*	0.09
BGC0002362	cyclic-lactone-autoinducer	Polyketide	loseolamycin A1, loseolamycin A2	*Micromonospora endolithica*	0.09
			**GKD7**		
BGC0002674	T3PKS	RiPP	enterocin F4-9	*Enterococcus faecalis*	0.23
BGC0000286	T3PKS	Polyketide	viguiepinol	*Streptomyces sp. KO-3988*	0.23
BGC0002561	T3PKS	Alkaloid	phenazine SA, phenazine SB, phenazine SC	*Streptomyces sp.*	0.19
BGC0002116	T3PKS	RiPP	darobactin A	*Photorhabdus khanii HGB 1456*	0.17
BGC0002382	T3PKS	RiPP	grimoviridin	*Grimontia marina*	0.16

BGC0001210	T3PKS	RiPP	pseudomycoicidin	*Bacillus pseudomycoides DSM 12442*	0.16
BGC0000551	T3PKS	RiPP	SapB	*Streptomyces coelicolor A3(2)*	0.16
BGC0002005	T3PKS	RiPP	RaxX	*Xanthomonas oryzae pv. oryzae*	0.16
BGC0000205	T3PKS	Polyketide	bryostatin	*Candidatus Endobugula sertula*	0.16
BGC0000590	T3PKS	RiPP	microcin N	*Escherichia coli*	0.16
BGC0000647	terpene	Terpene	carotenoid	*Rhodobacter sphaeroides*	0.45
BGC0000650	terpene	Terpene	carotenoid	*Algoriphagus sp. KK10202C*	0.32
BGC0000648	terpene	Terpene	carotenoid	*Myxococcus xanthus*	0.23
BGC0001456	terpene	Terpene	isorenieratene	*Streptomyces argillaceus*	0.21
BGC0001227	terpene	Terpene	isorenieratene	*Streptomyces collinus Tu 365*	0.21
BGC0000633	terpene	Terpene	carotenoid	*Streptomyces avermitilis*	0.21
BGC0000664	terpene	Terpene	isorenieratene	*Streptomyces griseus subsp. griseus NBRC 13350*	0.2
BGC0000630	terpene	Terpene	(2R,3S,3'S)-2-hydroxyastaxanthin	*Paracoccus haeundaensis*	0.2
BGC0000646	terpene	Terpene	β-carotein	*uncultured bacterium*	0.19
BGC0000645	terpene	Terpene	carotenoid	*Halobacillus halophilus DSM 2266*	0.19
BGC0002579	cyclic-lactone-autoinducer	RiPP	carnobacteriocin XY	*Carnobacterium maltaromaticum*	0.15
BGC0000811	cyclic-lactone-autoinducer	Alkaloid	fumigaclavine C	*Aspergillus fumigatus Af293*	0.12
BGC0000540	cyclic-lactone-autoinducer	RiPP	paenibacillin	*Paenibacillus polymyxa OSY-DF*	0.12
BGC0001573	cyclic-lactone-autoinducer	Alkaloid	dihydrolysergic acid	*Claviceps africana*	0.12
BGC0001267	cyclic-lactone-autoinducer	Terpene	lysergic acid, elymoclavine	*Claviceps fusiformis*	0.12
BGC0002667	cyclic-lactone-autoinducer	RiPP	estericin A	*Clostridium esterthеticum*	0.11

BGC0002362	cyclic-lactone-autoinducer	Polyketide	loseolamycin A1, loseolamycin A2	*Micromonospora endolithica*	0.11
BGC0000891	cyclic-lactone-autoinducer	Other (Aminocoumarin)	pentabromopseudilin	*Pseudoalteromonas luteoviolacea 2ta16*	0.1
BGC0001122	cyclic-lactone-autoinducer	NRP, Polyketide	dihydroisoflavipucine, isoflavipucine	*Aspergillus terreus NIH2624*	0.09
BGC0001521	cyclic-lactone-autoinducer	NRP	auriculamide	*Herpetosiphon aurantiacus DSM 785*	0.09
			**GKK1**		
BGC0000286	T3PKS	Polyketide	viguiepinol	*Streptomyces sp. KO-3988*	0.23
BGC0001387	T3PKS	Other	nucleocidin	*Streptomyces calvus*	0.2
BGC0002435	T3PKS	Other	coformycin, aristeromycin	*Micromonospora haikouensis*	0.2
BGC0001651	T3PKS	Other	legionaminic acid	*Tannerella forsythia 92A2*	0.19
BGC0002561	T3PKS	Alkaloid	phenazine SA, phenazine SB, phenazine SC	*Streptomyces sp.*	0.19
BGC0000769	T3PKS	Saccharide	glycopeptidolipid	*Mycobacterium avium subsp. hominissuis A5*	0.18
BGC0001617	T3PKS	Terpene	hirsutene	*Stereum hirsutum FP-91666 SS1*	0.17
BGC0002302	T3PKS	Saccharide	glucorhamnan	*[Ruminococcus] gnavus ATCC 29149*	0.16
BGC0000205	T3PKS	Polyketide	bryostatin	*Candidatus Endobugula sertula*	0.16
BGC0000913	T3PKS	Other	MK-8	*Enterobacter cloacae*	0.16
BGC0000647	terpene	Terpene	carotenoid	*Rhodobacter sphaeroides*	0.45
BGC0000650	terpene	Terpene	carotenoid	*Algoriphagus sp. KK10202C*	0.32
BGC0000645	terpene	Terpene	carotenoid	*Halobacillus halophilus DSM 2266*	0.25
BGC0000648	terpene	Terpene	carotenoid	*Myxococcus xanthus*	0.23
BGC0001456	terpene	Terpene	isorenieratene	*Streptomyces argillaceus*	0.21
BGC0001227	terpene	Terpene	isorenieratene	*Streptomyces collinus Tu 365*	0.21
BGC0000633	terpene	Terpene	carotenoid	*Streptomyces avermitilis*	0.21

BGC0000664	terpene	Terpene	isorenieratene	*Streptomyces griseus subsp. griseus NBRC 13350*	0.2
BGC0000646	terpene	Terpene	β-carotein	*uncultured bacterium*	0.19
BGC0000656	terpene	Terpene	zeaxanthin	*Xanthobacter autotrophicus Py2*	0.19
BGC0002579	cyclic-lactone-autoinducer	RiPP	carnobacteriocin XY	*Carnobacterium maltaromaticum*	0.15
BGC0000811	cyclic-lactone-autoinducer	Alkaloid	fumigaclavine C	*Aspergillus fumigatus Af293*	0.12
BGC0000540	cyclic-lactone-autoinducer	RiPP	paenibacillin	*Paenibacillus polymyxa OSY-DF*	0.12
BGC0001573	cyclic-lactone-autoinducer	Alkaloid	dihydrolysergic acid	*Claviceps africana*	0.12
BGC0001267	cyclic-lactone-autoinducer	Terpene	lysergic acid, elymoclavine	*Claviceps fusiformis*	0.12
BGC0002667	cyclic-lactone-autoinducer	RiPP	estericin A	*Clostridium estertheticum*	0.11
BGC0002362	cyclic-lactone-autoinducer	Polyketide	loseolamycin A1, loseolamycin A2	*Micromonospora endolithica*	0.11
BGC0000891	cyclic-lactone-autoinducer	Other (Aminocoumarin)	pentabromopseudilin	*Pseudoalteromonas luteoviolacea 2ta16*	0.1
BGC0001122	cyclic-lactone-autoinducer	NRP, Polyketide	dihydroisoflavipucine, isoflavipucine	*Aspergillus terreus NIH2624*	0.09
BGC0001521	cyclic-lactone-autoinducer	NRP	auriculamide	*Herpetosiphon aurantiacus DSM 785*	0.09

Table S3. Summary of bacteriocin gene clusters found in *L. plantarum* GKM3, GKK1, and

GKD7.

Strain	Type	Bit Score
GKM3	Enterocin X chain beta	50.83
	Plantaricin A	90.51
	Plantaricin F	107.07
	Plantaricin E	112.46
	Plantaricin J	42.74
GKD7	Enterocin X chain beta	50.45
	Plantaricin E	112.46
	Plantaricin F	107.07
GKK1	Plantaricin E	112.46
	Plantaricin F	105.15
	Plantaricin K	46.6
